# Supplementary material for: Getting More Out of Biomedical Documents with GATE's Full Lifecycle Open Source Text Analytics
Source: PLoS Comput Biol. 2013 Feb 7;9(2):e1002854. doi: 10.1371/journal.pcbi.1002854 (PMC3567135; doi:10.1371/journal.pcbi.1002854)
Supplement: Dataset S1 — GATE software. Dataset S1 bundled with this paper contains a distribution of GATE (or see http://gate.ac.uk/download/). (TGZ) [file pcbi.1002854.s001.tgz › plos-gate/plugins/Tagger_MetaMap/doc/serialized-form.html]

Serialized Form


---


|  |  |  |
| --- | --- | --- |
| **Overview** \| Package \| Class \| Use \| **Tree** \| **Deprecated** \| **Index** \| **Help** | |  |
| PREV   NEXT | **FRAMES**    **NO FRAMES**     **All Classes** |  |


---


# Serialized Form


---

**Package** **gate.metamap**

**Class gate.metamap.MetaMapPR extends gate.creole.AbstractLanguageAnalyser implements Serializable**

**Serialized Fields**

### inputASName

```
java.lang.String inputASName
```

---

### inputASTypes

```
java.util.ArrayList<E> inputASTypes
```

---

### inputASTypeFeature

```
java.lang.String inputASTypeFeature
```

---

### outputASName

```
java.lang.String outputASName
```

---

### outputASType

```
java.lang.String outputASType
```

---

### outputMode

```
OutputMode outputMode
```

---

### annotatePhrases

```
java.lang.Boolean annotatePhrases
```

---

### metaMapOptions

```
java.lang.String metaMapOptions
```

---

### annotateNegEx

```
java.lang.Boolean annotateNegEx
```

---

### linebreakCount

```
java.lang.Integer linebreakCount
```

---

### taggerMode

```
TaggerMode taggerMode
```

---


|  |  |  |
| --- | --- | --- |
| **Overview** \| Package \| Class \| Use \| **Tree** \| **Deprecated** \| **Index** \| **Help** | |  |
| PREV   NEXT | **FRAMES**    **NO FRAMES**     **All Classes** |  |


---
